# Accuracy of GE digital breast tomosynthesis versus supplementary mammographic views for diagnosis of screen-detected soft tissue breast lesions

**DOI:** 10.1186/bcr3757

**Published:** 2015-11-05

**Authors:** Eleanor Cornford, Anne Turnbull, Jonathan James, Rachel Tsang, Tayeba Akram, Helen Burrell, Lisa Hamilton, Sarah Tennant, Mark Bagnall, Shama Puri, Graham Balls, Yan Chen, Vivienne Jones

**Affiliations:** 1 Nottingham Breast Institute, Nottingham, UK; 2 Royal Derby Hospital, Derby, UK; 3 Nottingham Trent University, Nottingham, UK; 4 Loughborough University, Loughborough, UK; 5 Northampton General Hospital, Northampton, UK

## Introduction

The aim was to compare the accuracy of standard supplementary views and GE digital breast tomosynthesis (DBT) for assessment of soft tissue mammographic abnormalities.

## Methods

Women recalled for further assessment of soft tissue abnormalities were recruited and received standard supplementary views (typically spot compression views) and two-view GE DBT. The added value of DBT in the assessment process was determined by analysing data collected prospectively by unblinded radiologists working up the cases.

Following anonymisation of cases, there was also a retrospective multireader review. The readers first read bilateral standard two-view digital mammography (DM) together with the supplementary mammographic views and gave a combined score for suspicion of malignancy on a five-point scale. The same readers then read bilateral standard two-view DM together with two-view DBT. Pathology data were obtained. Differences were assessed using ROC analysis.

## Results

The study population was 342 lesions in 322 patients. Final diagnosis was malignant in 113 cases (33%) and benign/normal in 229 cases (67%). In the prospective analysis, the performance of two-view DM plus DBT was at least equivalent to the performance of two-view DM and standard mammographic supplementary views--area under the curve (AUC) was 0.946 and 0.922 respectively, which did not reach statistical significance. Similar results were obtained for the retrospective review--AUC was 0.900 (DBT) and 0.873 (supplementary views), which did not reach statistical significance.

## Conclusion

The accuracy of GE DBT in the assessment of screen-detected soft tissue abnormalities is equivalent to the use of standard supplementary mammographic views.

